# Parental Optimism and Perceived Control over Children’s Initiation of Tobacco, Cannabis, and Opioid Use

**DOI:** 10.3390/ijerph17176181

**Published:** 2020-08-26

**Authors:** Nicholas Chadi, Jonathan P. Winickoff, Olivier Drouin

**Affiliations:** 1 Sainte-Justine University Hospital Centre, Division of Adolescent Medicine, Department of Pediatrics, University of Montreal, Montreal, QC H3T 1C5, Canada; 2 Massachusetts General Hospital, Department of Pediatrics, Harvard Medical School, Boston, MA 02114, USA; jwinickoff@mgh.harvard.edu; 3 Sainte-Justine University Hospital Centre, Division of General Pediatrics, Department of Pediatrics, University of Montreal, Montreal, QC H3T 1C5, Canada; o.drouin@umontreal.ca

**Keywords:** behavior control, cannabis, opioids, optimism, parents, smoking

## Abstract

Families play an important role in helping teenagers avoid using tobacco, cannabis, and opioids, but some parents may underestimate the risk of their children using those substances. This study aimed to determine parents’ perceived likelihood of their child initiating tobacco, cannabis, and opioid use, as well as the control they have in preventing their child from using those substances. We surveyed 427 parents of children aged 0–18 years old using the online Amazon Mechanical Turk platform in the spring of 2019. We measured participants’ perceived likelihood of their child initiating tobacco, cannabis, or opioid use before the age of 18 compared to other children, using a five-point Likert scale. This perceived likelihood was dichotomized between optimistic (less likely than average) and non-optimistic (average or more likely than average). Independent variables included parental tobacco use, perceived parental control, and perceived severity of the behavior. Participants with missing data and participants with children who had already initiated substance use were excluded from statistical analyses. Mean age of participants was 38.1 years (Standard Deviation 8.4); 67% were female. Level of parental optimism was 59% for cannabis, 77% for tobacco, and 82% for opioids. Perceived severity was significantly lower for cannabis use (71/100) than tobacco (90/100) and opioid use (92/100) (*p* < 0.001). Current smokers were less likely than never smokers to be optimistic about their child’s risk of initiating using tobacco (Adjusted Odds Ratio (AOR): 0.18 [95% Confidence Interval (CI) 0.10–0.34]) or cannabis (AOR: 0.21 [95% CI 0.12–0.38]). Parental perceived likelihood of a child initiating substance use represents an understudied and potential target for substance use prevention.

## 1. Introduction

The nearly six-fold reduction in rates of cigarette smoking among youth in countries like Canada and the USA in the past three decades represents one of the greatest public health success stories of our time [1,2]. Population-level education campaigns that increased perceptions of harm from cigarette use have played an important role in this reduction [3]. While levels of perceived harm have increased for tobacco and opioids, trends in perceived harm among youth and their parents vary substantially across substances [4]. For instance, in parallel with increasing legality and availability of cannabis, youth and parental perceptions of harm associated with cannabis use are reaching a record low [5]. In turn, this shift has been associated with a steady increase in rates of daily cannabis use and use of higher potency cannabis products among North American youth [1,2]. 

Parents can have a major influence on their children’s use of addictive substances. In fact, parental attitudes and behaviors related to addictive substances during their children’s perinatal, early childhood, and adolescent development are associated with increased rates of substance use disorders during adolescence [6]. In this sense, improving understanding of parental perceptions around substance use could help design effective risk communication strategies and improve primary prevention of substance use in children and adolescents. For example, authoritative parenting styles have been associated with lower rates of adolescent alcohol, tobacco, and cannabis use [7]. As such, adolescents who perceive that their parents would respond negatively and be upset by their use of cigarettes are less likely to use them [8]. Conversely, overly permissive or restrictive parenting practices related to substance use, as well as parental substance use itself, represent strong predictors of adolescent use of both licit and illicit drugs [9,10]. 

Perceived riskiness is an important component of decision making and represents an important protective factor for the prevention of substance use in adolescents [11,12]. The impact of parental perceptions of riskiness of their child’s use of substances remains poorly understood, but represents an important potential target for interventions. In a recent study, most parents surveyed believed their children were at lower risk than the average child of initiating cigarette smoking [13]. High levels of optimism, also known as optimistic bias, have also been shown for other chronic pediatric health conditions [13,14].

Parental perceived control over a child’s behavior is a construct built on an internal parental resource [15]. Perceived control can be divided into two dimensions: (1) proximal control, which refers to a parent’s perceived level of control over exercising a specific behavior (e.g., a specific parenting behavior); and (2) distal control, which pertains to a longer-term view of a parent’s ability to affect a child’s behavior (e.g., initiating substance use) [16]. The relationship between optimistic bias and level of perceived control has been described thus: in most cases, increased perceived control leads to increased optimism [17]. However, the extent to which this relationship may apply to parental perceptions of their child’s risk of using substances like tobacco, cannabis, and opioids remains unknown.

This is the first study to compare levels of parental optimism and perceived control of their children’s use of tobacco, cannabis, and opioids before the age of 18. We hypothesized that the level of optimism would be high for all three substances and that respondents who were current smokers would report lower levels of optimism and parental control over their child’s substance use than respondents who had never smoked before. We based this second hypothesis on previous literature showing that youth with parents who are current or former smokers are at increased risk of using substances during adolescence [6]. 

## 2. Materials and Methods 

### 2.1. Study Design

This study was a one-time cross-sectional online survey. Survey questions were selected based on a review of the existing literature and build on existing work from senior authors O.D. and J.P.W. [13,18].

### 2.2. Participants

Participants were recruited between January and May 2019 through the online Amazon Mechanical Turk crowdsourcing platform [19]. This platform allows rapid access and recruitment among a large pool of potential participants. It is increasingly being used for online surveys and has been found to produce reliable and reproducible results at a low cost for study investigators [19,20]. To be eligible to participate in the study, interested individuals needed to be 18 years or older, be the parent of at least one child, live in Canada or the USA, and have sufficient English language fluency to complete the study survey. Potential participants whose children were 18 years old or older were excluded from the study. 

### 2.3. Procedure

After signing an online consent form, participants were directed to the secured Research Electronic Data Capture (REDCap) platform to complete an online survey. Each participant received a small monetary compensation ($0.50 USD) for completing the survey, and the survey took approximately 10 min to complete. The study protocol received approval from the institutional review board of Sainte-Justine University Hospital Centre, Montreal, QC (reference number 2019–2139).

### 2.4. Outcomes

#### 2.4.1. Primary Outcome

The primary outcome for this study was parental perceived risk of their children initiating use of each of the three following substances: tobacco, cannabis, or opioids before the age of 18. If the participant had more than one child, the survey asked them to answer for their oldest child (who was 18 years or younger). Level of relative perceived risk was measured using a 5-point Likert scale, from “much less likely” to “much more likely”, similar to previous studies on this topic [13,21]. Specifically, participants were asked: “Compared to other children of his/her age, how likely is your child to use [tobacco/cannabis/opioids] before age 18?” Participants were considered “optimistic” if they believed their child was “less likely” or “much less likely” than other children of the same age to develop each outcome. Conversely, participants were considered “non-optimistic” if they thought their child was either “as likely”, “more likely”, or “much more likely” compared to other children to develop each outcome. It was decided to dichotomize participants at a threshold that would allow meaningful statistical inferences while providing similar sized groups, consistent with previous literature [13,14].

#### 2.4.2. Secondary Outcomes

Perceived control was measured using a 5-point Likert scale (from 0 to 4). Participants were asked: “How much control do you think a parent has in preventing their child from using [tobacco/cannabis/opioids]”, with possible answers ranging from “no control at all” (0/4) to “a great deal of control” (4/4). Perceived severity of substance use was measured using a 100-point scale ranging from 1 (a very minor health problem such as dandruff) to 100 (a likely fatal disease such as incurable cancer).

### 2.5. Statistical Analyses

We used descriptive statistics to report participant and child characteristics and describe levels of parental optimism for each of the three substances under study (tobacco, cannabis, and opioids). When participants reported having multiple children under the age of 18, child characteristics were those of the participant’s oldest child. We compared perceived risk of uptake for each of the three substances using a Chi Square test. We conducted multivariable logistic regression analyses using parental optimism for their child’s use of the three substances as the dichotomous outcomes of interest among parents whose child had not yet initiated substance use. Regression analyses were also conducted to assess differences in perceived control over participants’ children’s risk of using substances. Participants with missing data and participants with a child who had already initiated use of any of the three substances were excluded from optimism, perceived control, and disease severity analyses, as this was perceived to be an important confounding factor (in total: 27 participants (13%)). All regression models were adjusted for participant gender, educational achievement (as an indicator of socio-economic status), age category of the child (teenager vs. not), and respondent smoking status. All statistical analyses were carried with SAS 9.4 statistical software (Cary, NC) and used the standard threshold of *p* < 0.05 for statistical significance. 

## 3. Results

### 3.1. Participant Characteristics

In total, 427 respondents completed the survey. Mean participant age was 38.1 (Standard Deviation (SD) 8.4) years, and approximately two-thirds (67%) were female. The majority had completed a college or graduate degree (63%). Mean age of participants’ children was 9.3 years (SD 5.1), and half of the children were girls (50%). Participant characteristics are summarized in Table 1. 

### 3.2. Parental Perceived Risk, Control, and Severity

**Figure 1:**
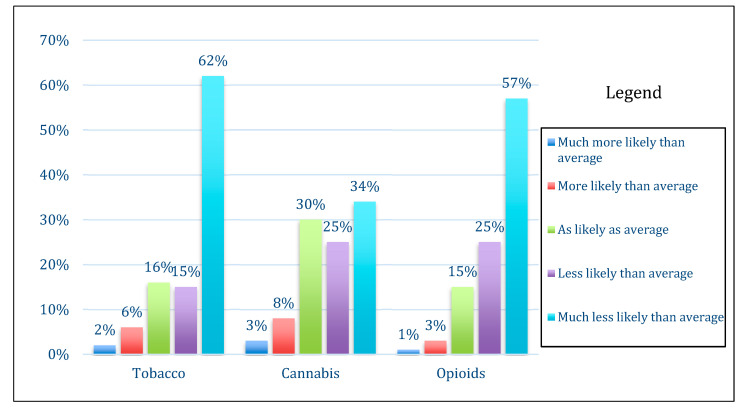
Distribution of parental assessment of likelihood of child’s risk of tobacco, cannabis, and opioid use initiation before age 18 (*n* = 400). Participants with missing data and participants with children who had already initiated smoking, cannabis, or opioid use were excluded from optimism analyses.

Overall, among participants with complete data and whose children had not yet initiated substance use (*n* = 400), the majority were optimistic about their child’s future avoidance of tobacco, cannabis, or opioids before the age of 18. There were significant differences in parental optimism across the three substances, with the majority of participants estimating that their child was much less likely than the average child to use tobacco (62%) and opioids (57%) but only a minority of participants (34%) estimating that their child was much less likely than average to use cannabis (*p* < 0.001). Distribution of parental risk perception for the three substances is shown in Figure 1. Perceived severity was significantly lower for cannabis use (71/100, SD 31) than for tobacco (90/100, SD 15) and opioid use (92/100, SD 14) (*p* < 0.001). Overall, there were no differences in perceived parental control (on a four-point scale) between the three substances (tobacco: 2.67/4, cannabis: 2.65/4, opioids: 2.84/4; *p* = 0.21).

Multivariable logistic regression analyses revealed that current smokers and former smokers had lower optimism regarding their child’s use of tobacco when compared to never smokers as shown in Table 2.

There were no significant differences between male and female respondents and between participants with and without a college degree regarding optimism about their child’s intention to use substances. Current smokers reported lower control regarding their child’s use of cigarettes, but not cannabis and opioids when compared to never smokers as shown in Table 3. On the other hand, former smokers reported lower control regarding their child’s use of all three substances when compared to never smokers.

## 4. Discussion

Our study found that the majority of participants reported high levels of optimism regarding their child’s future avoidance of tobacco, cannabis, and opioids. While optimism was significantly lower for cannabis use compared with tobacco and opioid use, there were no significant differences in perceived level of parental control between the three substances. Our results also revealed that participants who were current and former smokers had lower optimism about their child’s future use of tobacco and cannabis than never smokers, and that current and former smokers endorsed lower levels of parental control over use of tobacco when compared with never smokers. Our results are consistent with a previous study by Drouin and colleagues that found high levels of parental optimism with regards to their child’s future risk of initiating smoking, yet, lower levels of optimism among parents who reported there was a smoker in the home [13]. To our knowledge, no other study has assessed parental optimism with regards to their child’s future use of cannabis and opioids. 

Parental optimism in the context of chronic medical conditions has been shown to be a helpful component of treatment and recovery [22]. However, when it comes to adolescent substance use, optimism may carry a different significance. In fact, the human brain continues to develop well into the third decade of life. Until it is fully mature, the adolescent brain is biologically primed to seek rewarding sensations such as those brought by using tobacco, cannabis, or opioid drugs [23]. This evolutionary programming can help explain the vulnerability of adolescents to initiating substance use and developing substance use disorders [24,25]. The high levels of optimism seen in our study suggest that most parents may believe that their child’s risk of initiating substance use before age 18 is low. These same parents may then spend less time and energy trying to minimize the chances of their children initiating substance use during adolescence. 

The differences in optimism and perceived parental control that we found between never smokers and past/current smokers suggest that parents’ past substance use history has an impact on how they may approach this topic with their children. Current and former smokers are likely to have experienced some of the negative effects of substance use first-hand. Considering that youth who have used cigarettes are likely to engage with parents about smoking [26], there is a need to help parents build on their lived experience and feel empowered to guide their children towards healthier behavioral choices. To the extent that some parents might underestimate their child’s risk of tobacco, cannabis, or opioid use, parental education on the actual risk might increase the attention parents place on use prevention. The lower levels of parental optimism seen in past/current smokers can be interpreted as increased realism. However, the lower level of perceived control among former smokers also raises the question of whether former smokers believe that preventing their child from using those substances may be linked to factors that are beyond their control (i.e., genetic factors). 

This study has several strengths and implications. As the first study to look at parental optimism and control over children’s future use of tobacco, cannabis, and opioids together, it could help stimulate thinking about future interventions that might address paths to adolescent polysubstance use and addiction. Given aggressive marketing strategies coming from the tobacco and cannabis industries, which tend to minimize negative consequences related to the use of those substances [27], our study may help guide the design of effective clinical and public health interventions to reduce or delay onset of substance use among youth. As a logical next step to our work, future longitudinal studies could also help assess the relationship between parental perceptions and their child’s future use of substances as youth age through adolescence and young adulthood. 

Study results should be interpreted considering certain limitations. First, participant recruitment took place voluntarily through an openly accessible online platform, introducing a risk of selection bias, limiting the external validity, and potentially affecting the generalizability of our findings. Unfortunately, information about respondents’ race/ethnicity, marital status, and use of substances other than tobacco was not available. Second, data were collected by self-report from parents only, introducing the risk of reporting bias about parental smoking and child initiation of substance use. Third, while comparison of risk perception within child-parent dyads would have been interesting, the format of the survey and the platform used precluded us from obtaining data from children and conducting this analysis. Finally, due to the low incidence of substance use reported in participants’ children, and due to the fact that many participants had very young children, it was not possible to account for the effect of children’s substance use on parental perceptions of risk and control. 

## 5. Conclusions

Perceptions of risk and control over children’s use of tobacco, cannabis, and opioids represent an understudied and potential target for substance use prevention in adolescents. Although our study was not designed to measure the impacts of parental optimism and perceived parental control over their child’s initiation of substance use, it is well known that higher perceptions of risk and authoritative parenting styles are associated with decreased rates of initiation of use of psychoactive substances among adolescents. Increasing parental levels of concern about adolescent substance use and encouraging them to share those concerns with their children may represent a promising strategy to increase perceptions of risk among youth. To maximize effectiveness, adolescent substance use prevention efforts should employ multi-pronged approaches, combining both individual-level and population-level strategies [28]. In the context of rapidly evolving epidemiological and policy landscapes, there is a great need for youth to be informed by trusted sources, including parents, about the potential harms of adolescent substance use.

## Figures and Tables

**Table 1:** Participant and child characteristics ^1^.

Characteristics	*n* (%) ^a^ or Mean (SD) ^b^	Missing ^2^
Total	*n* = 427	
Parent’s characteristics		
Age (years) ^b^	38.1 (8.4)	20
Female ^a^	274 (67.3)	20
Education ^a^		21
Elementary/Some high school/High school graduate	35 (8.6)	
Some college	117 (28.8)	
College graduate	191 (47.0)	
Graduate degree	63 (15.5)	
Smoking ^a^		12
Smoker in the house	93 (22.4)	
Respondent smokes cigarettes everyday	58 (14.0)	
Respondent smokes cigarettes some days	18 (4.3)	
Respondent is a former smoker	123 (29.6)	
Respondent has never smoked cigarettes	216 (52.0)	
		
Child’s characteristics ^3^		
Child age (years) ^b^	9.3 (5.1)	1
Female ^a^	203 (50.0)	21
Teenager: 13–17 years old ^a^	140 (32.9)	1
Substance use ^a^		
Child has consumed cannabis (yes)	8 (6.0) ^4^	6
Child has used opioids (yes)	2 (1.5) ^4^	5
Child smokes cigarettes (yes)	1 (0.7) ^4^	0

^1^ Percentages for participants with non-missing data. ^2^ In total, 8 participants had children who had already used one or many substances, and 21 participants had missing data on at least one study question. For participants who reported having more than one child under the age of 18, characteristics are those of the oldest child. ^3^ Percentage among teenagers (age 13–17 years) only. ^4^ Parent reported child lifetime use. We use the superscript “^a^” for characteristics for which number of respondents and % are reported and the superscript “^b^” for characteristics for which mean value and standard deviation are reported. Abbreviation: SD: Standard Deviation.

**Table 2:** Parental likelihood of being optimistic over their child’s use of tobacco, cannabis, and opioids before age 18 (*n* = 400) ^1^.

Respondent Characteristics	Optimism ^2^: Tobacco	Optimism: Cannabis	Optimism: Opioids
	AOR ^3^ (95% CI)	AOR (95% CI)	AOR (95% CI)
Men (vs. women)	1.00 (0.59, 1.72)	1.36 (0.86, 2.15)	1.46 (0.81, 2.64)
College graduate (vs. not)	1.10 (0.65, 1.86)	1.31 (0.84, 2.04)	0.90 (0.51, 1.58)
Parent of teenager (vs. parent of younger child)	1.04 (0.61, 1.77)	1.19 (0.75, 1.87)	1.19 (0.67, 2.11)
Current smoker (vs. Never smoker)	**0.18 (0.10, 0.34)**	**0.21 (0.12, 0.38)**	0.59 (0.30, 1.16)
Former smoker (vs. Never smoker)	**0.47 (0.26, 0.85)**	**0.47 (0.29, 0.76)**	1.09 (0.58, 2.06)

^1^ Participants with missing data and participants with children who had already initiated smoking, cannabis, or opioid use were excluded from optimism analyses. ^2^ Optimism represents the likelihood that a parent reports their child is less likely or much less likely than the average child to start using substances before age 18. ^3^ All regression models were adjusted for participant gender, educational achievement (as a measure of socio-economic status), age category of the child (teenager vs. not), and respondent smoking status. Statistically significant values (*p* < 0.05) are bolded. Abbreviations: AOR: Adjusted Odds Ratio; CI: Confidence Interval.

**Table 3:** Difference in perceived parental control over their child’s initiation of tobacco, cannabis, and opioid use (*n* = 400) ^1^.

Respondent Characteristics	Tobacco	Cannabis	Opioids
	Delta ^2^ (95% CI)	Delta (95% CI)	Delta (95% CI)
Men (vs. women)	0.17 (−0.06, 0.40)	0.09 (−0.14, 0.33)	0.12 (−0.11, 0.36)
College graduate (vs. not)	−0.09 (−0.32, 0.13)	−0.08 (−0.32, 0.15)	−0.19 (−0.42, 0.04)
Parent of teenager (vs. parent of younger child)	−0.22 (−0.45, 0.01)	−0.19 (−0.43, 0.04)	−0.16 (−0.40, 0.07)
Current smoker (vs. Never smoker)	**−0.34 (−0.63, −0.04)**	−0.16 (−0.46, 0.15)	−0.16 (−0.46 0.14)
Former smoker (vs. Never smoker)	**−0.32 (−0.56, −0.07)**	**−0.31 (−0.56, −0.05)**	**−0.26 (−0.52, −0.01)**

^1^ Participants with missing data and participants with children who had already initiated smoking, cannabis, or opioid use were excluded from perceived control analyses. ^2^ Deltas correspond to the mean difference in perceived control between the two comparison groups adjusted for participant gender, educational achievement (as a measure of socio-economic status), age category of the child (teenager vs. not), and respondent smoking status. Perceived control scores represent participants’ answer to the question: “How much control do you think a parent has in preventing their child from using tobacco/cannabis/opioids” reported on a 5-point Likert scale (range: 0–4). Statistically significant values (*p* < 0.05) are bolded. Abbreviation: CI: Confidence Interval.
